# Dynamic Knee Joint Line Orientation Is Not Predictive of Tibio-Femoral Load Distribution During Walking

**DOI:** 10.3389/fbioe.2021.754715

**Published:** 2021-11-02

**Authors:** Adam Trepczynski, Philippe Moewis, Philipp Damm, Pascal Schütz, Jörn Dymke, Hagen Hommel, William R. Taylor, Georg N. Duda

**Affiliations:** ^1^ Berlin Institute of Health at Charité – Universitätsmedizin Berlin, Julius Wolff Institute, Berlin, Germany; ^2^ Institute for Biomechanics, ETH Zürich, Zürich, Switzerland; ^3^ Krankenhaus Märkisch-Oderland, Wriezen, Germany; ^4^ Medizinische Hochschule Brandenburg, Theodor Fontane, Neuruppin, Germany

**Keywords:** total knee arthroplasty, in vivo loading, mobile fluoroscopy, knee joint line, external adduction moment, medio-lateral knee load distribution

## Abstract

Some approaches in total knee arthroplasty aim for an oblique joint line to achieve an even medio-lateral load distribution across the condyles during the stance phase of gait. While there is much focus on the angulation of the joint line in static frontal radiographs, precise knowledge of the associated dynamic joint line orientation and the internal joint loading is limited. The aim of this study was to analyze how static alignment in frontal radiographs relates to dynamic alignment and load distribution, based on direct measurements of the internal joint loading and kinematics. A unique and novel combination of telemetrically measured *in vivo* knee joint loading and simultaneous internal joint kinematics derived from mobile fluoroscopy (“CAMS-Knee dataset”) was employed to access the dynamic alignment and internal joint loading in 6 TKA patients during level walking. Static alignment was measured in standard frontal postoperative radiographs while external adduction moments were computed based on ground reaction forces. Both static and dynamic parameters were analyzed to identify correlations using linear and non-linear regression. At peak loading during gait, the joint line was tilted laterally by 4°–7° compared to the static joint line in most patients. This dynamic joint line tilt did not show a strong correlation with the medial force (*R* ^2^: 0.17) or with the mediolateral force distribution (pseudo *R* ^2^: 0.19). However, the external adduction moment showed a strong correlation with the medial force (*R* ^2^: 0.85) and with the mediolateral force distribution (pseudo *R* ^2^: 0.78). Alignment measured in static radiographs has only limited predictive power for dynamic kinematics and loading, and even the dynamic orientation of the joint line is not an important factor for the medio-lateral knee load distribution. Preventive and rehabilitative measures should focus on the external knee adduction moment based on the vertical and horizontal components of the ground reaction forces.

## Introduction

**FIGURE 1:**
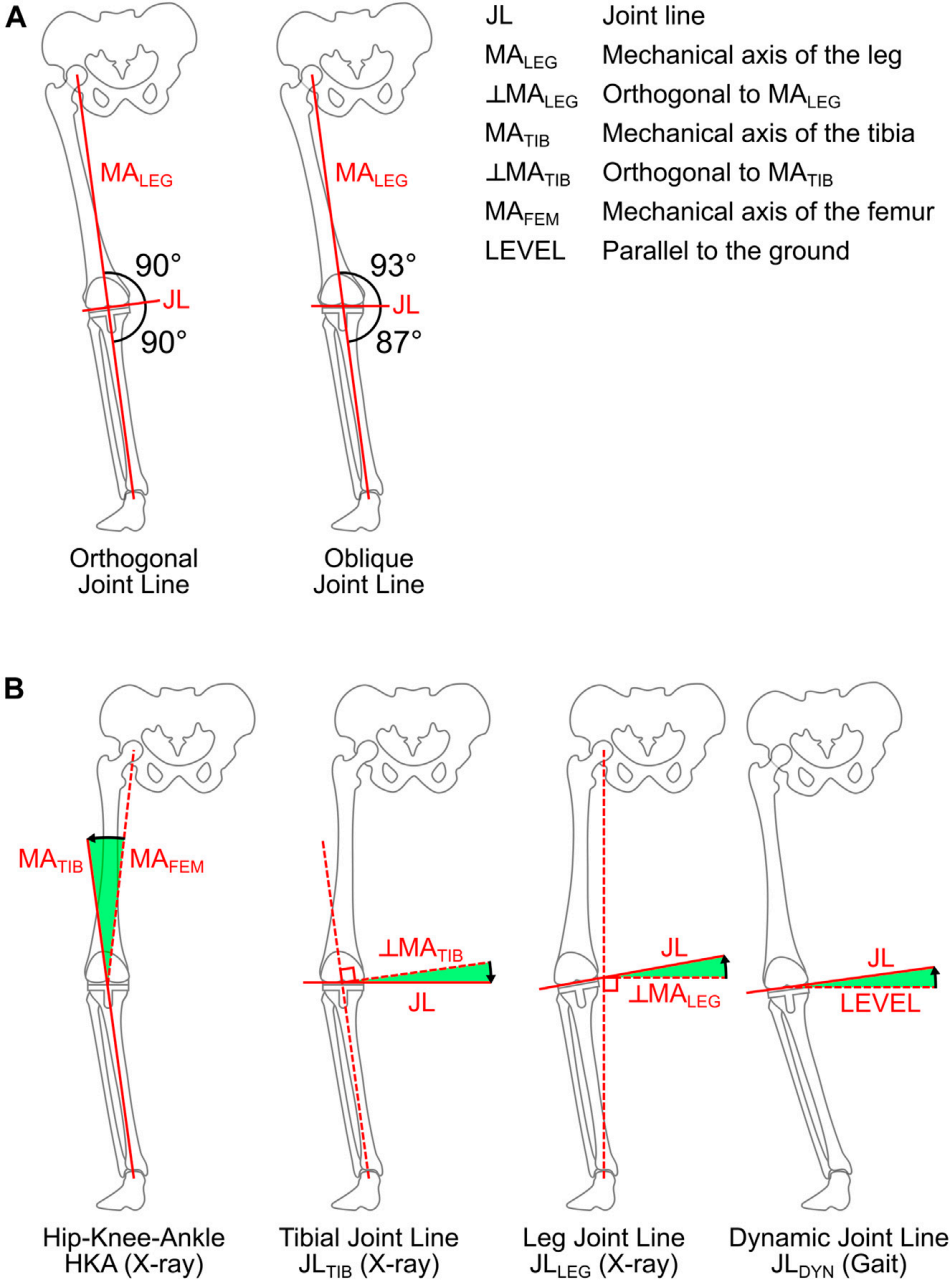
Orthogonal and oblique joint line alignment relative to the mechanical axis of the leg during loaded single leg support with the leg axis leaning laterally **(A)**. Parameters used to quantify the alignment in static frontal plane radiographs and from fluoroscopic reconstruction during gait **(B)**. The black arrow indicates the positive direction for each angle.

In Total Knee Arthroplasty (TKA), frontal plane geometry is usually determined using static standing radiographs, where the alignment is mainly quantified by: 1) the relative orientation of the femoral and tibial mechanical axes (varus-valgus of the leg) and 2) the orientation of the joint line to the mechanical axes. These measures are used to infer the mechanical conditions in the joint, and form the basis for different alignment techniques in knee joint reconstruction. For decades, the standard implantation approach in orthopedics has been to use the Mechanical Alignment Technique (MAT), which aims for a joint line that is orthogonal to a neutral mechanical axis (varus-valgus of zero). While the long-term implant survivorship of MAT is generally good (Patil et al., 2015), it doesn’t consistently lead to satisfactory functional outcomes (Nam et al., 2014), which is generally attributed to the often substantial alterations of the pre-operative leg geometry and associated overloading of the surrounding soft tissues (Bellemans et al., 2012; Gu et al., 2014; Hosseini Nasab et al., 2020). Alternative techniques involving a joint line that is oblique to the mechanical axis, like Anatomical Alignment Technique (AAT) (Hungerford et al., 1982) and Kinematic Alignment Technique (KAT) (Howell and Hull, 2014), have been proposed to address the issues of MAT. One rationale behind an oblique joint line is that during the single leg support phase of gait, the mechanical axis of the leg is assumed to tilt laterally, aligning the oblique joint line parallel to the ground (Figure 1A). Here, a more horizontal joint line in the loaded stance phase of gait is assumed to result in a more symmetrical distribution of the contact loads between the medial and lateral compartments of the knee (Riviere et al., 2017a), which is thought to be beneficial for the survivorship of TKA (Andriacchi et al., 1986). However, the actual dynamic joint line kinematics and the corresponding knee loading have so far not been directly measured *in vivo* during gait.

So far, it is—to the best of our knowledge—unknown how the joint line orientation in static frontal radiographs relates to the dynamic joint line during gait. In particular it is not clear whether the widely accepted AAT recommendation of a 3° medial tilt of the joint line relative to the mechanical axis, actually leads to a horizontal joint line relative to the ground at the instant of highest knee joint loading. It is also unknown if a horizontal dynamic joint line would actually lead to a more even load distribution between the medial and lateral condyles, as suggested by the AAT motivation. The aim of this study was therefore to analyze 1) how static alignment relates to the dynamic alignment during the stance phase of gait, and 2) how static and dynamic alignment relates to dynamic joint loading *in vivo* during gait.

Regarding the kinematics, we hypothesized that the difference between the static joint line (relative to the mechanical axis) and the dynamic joint line orientation (relative to the ground) would be at least 3°, implying that a 2°–3° oblique static joint line is more horizontal dynamically than an orthogonal static joint line. Regarding the knee contact loading, we hypothesized that the dynamic medio-lateral joint load distribution would correlate with the dynamic joint line orientation (relative to the ground).

## Materials and Methods

### Patients and Measurements

Six patients (5 male, 1 female), who had previously undergone primary TKA for osteoarthritis were recruited for this study. The patients were aged 74 (65–80) [mean (range)] years, had body-mass of 89 (67–101) kg, and a body-height of 172 (165–175) cm (Table 1). The patients were implanted with an instrumented TKA which allows the telemetric measurements of *in vivo* tibio-femoral contact forces and moments, and is based on the posterior cruciate sacrificing (PCS) INNEX-FIXUC system (Zimmer, Switzerland) (Heinlein et al., 2007). The FIXUC design features a symmetrical, ultra-congruent PE inlay, of which all patients received the same size M/10. All surgeries were performed in a single institution by two experienced surgeons using a medial parapatellar approach and the tibia-first gap-balancing technique, with all components being cemented.

**TABLE 1:** Patients: The anthromorphic data of at the time of the measurement, pre-operative Knee Society Score, the pre-operative and post-operative hip-knee-ankle (HKA) angles.

Patient	Gender	Age [years]	Body height [cm]	Body mass [kg]	Pre-operative knee society score	Pre-operative HKA angle [°]	Post-operative HKA angle [°]
K1L	m	70	175	101	65	8	3
K2L	m	78	169	91	75	8	5
K3R	m	77	173	100	63	9	3.5
K5R	m	65	174	96	90	11	1
K7L	f	80	165	67	70	15	6.5
K8L	m	76	175	79	95	11	4
MEAN		74.3	171.8	88.9	76.3	10.3	3.8

The following parameters were determined in postoperative standing frontal radiographs: the varus of the entire leg based on the hip-knee-ankle angle (HKA), the varus of the tibial implantation (JL_TIB_) based on the angle of the joint line to the tibial axis, and the mechanical joint line (JL_LEG_) based on the angle of the joint line to the orthogonal of the mechanical axis of the leg (hip-ankle-line) (Figure 1B). The JL_LEG_ also represents the lateral tilt angle of the joint line to the horizontal in the frontal plane, if the mechanical axis of the leg is vertical.

The synchronized assessment of the 3D kinematics and kinetics for this specific cohort has been published previously (Taylor et al., 2017; Trepczynski et al., 2019), and is herein only briefly summarized: A gait analysis was performed 5–7 years post-operatively, during which each patient performed four to five repetitions of a level walking activity, resulting in six to nine gait cycles available for analysis per patient. During gait, internal TF kinematics were captured using a mobile video-fluoroscope (List et al., 2017), while TF contact loading was synchronously measured using an instrumented tibial component (Heinlein et al., 2007).

### Quantification of Kinematics and Loading

**FIGURE 2:**
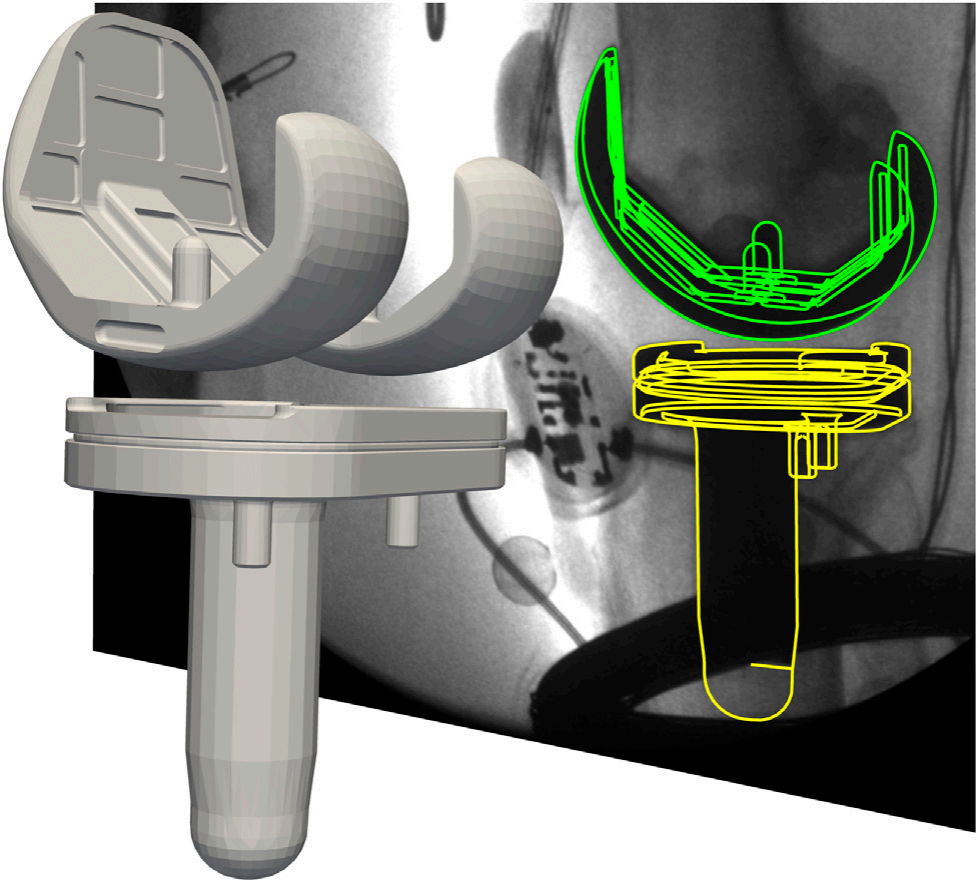
Reconstructed 3D tibio-femoral kinematics alongside with the corresponding mobile fluoroscopy image. The green and yellow lines represent the projection of the CAD-models onto the image plane.

The 3D positions and rotations of the implant components in the laboratory coordinate system were reconstructed from the fluoroscopic images (Figure 2), using a methodology already validated for a similar TKA design in an earlier investigation (List et al., 2017), which reported the rotational/translational errors of the 3D reconstruction as 0.15°/0.3 mm in plane, and 0.25°/1 mm out of plane. The 3D rotation of the tibial component in the laboratory coordinate system was then used to determine the lateral tilt angle of the joint line to the horizontal in the frontal plane (JL_DYN_) (Figure 1B). The *in vivo* measurement of the tibio-femoral loading was used to derive the medial and lateral axial force components F_MED_, F_LAT_ as previously described for the same telemetric implant by Kutzner et al. (Kutzner et al., 2017). The medio-lateral force distribution was quantified by the medial force ratio (MFR)

$$\mathrm{MFR}\;=\;F_{\mathrm{MED}}\text{/}\left(F_{\mathrm{MED}}+\;F_{\mathrm{LAT}}\right)$$

where F_MED_ + F_LAT_ represents the total axial contact force.

The external knee moment (EKM) was computed using the inverse dynamics approach (Andrews, 1974; Deuretzbacher and Rehder, 1995), based on kinematics that where determined using a combination of functional methods (Taylor et al., 2010; Ehrig et al., 2011; Heller et al., 2011), and a global optimization approach (Trepczynski et al., 2012). Reported forces were normalized to the patient’s body-weight (BW), while moments were normalized to the patient’s body-weight times body-height (BWHt).

### Statistical Analysis

Linear regressions performed within the R-software v4.0.2 (R-Core-Team, 2017) were used to the relate static and dynamic alignment, as well as external loads to internally measured loads. Since the MFR is bound between 0 and 1, a linear model was not appropriate. Therefore a non-linear model of the following form was fitted to the data (Trepczynski et al., 2014):

$$y\;=\;\arctan\left(b_{1}\;\text{*}\;x\;-\;b_{2}\right)\text{/}\;\pi \;+\;\text{0}\text{.}5$$

The quality of the non-linear fit was assessed using the Cox and Snell pseudo *R* ^2^ measure as implemented in the “rcompanion” package (version 2.3.26) of R-software (R-Core-Team, 2017).

## Results

### Static vs Dynamic Joint Line Alignment

In the static frontal radiographs, the patients had a wide range of tibio-femoral mechanical axis alignments, with HKA values between 1° and 6.5° (mean: 3.8°). Similarly, the tibial implantation varus JL_TIB_ varied between 0.4° and 3.9° (mean: 1.8°). However, the joint lines were mostly orthogonal to the mechanical axis of the leg (hip-ankle-line), with JL_LEG_ values between −0.3° and 1.3° (mean: −0.2°). In summary, while the patients were not neutrally aligned in terms of tibio-femoral mechanical axes, the joint lines were still orthogonal to the hip-ankle-line (except for patient K3R who had a small lateral joint line tilt).

**FIGURE 3:**
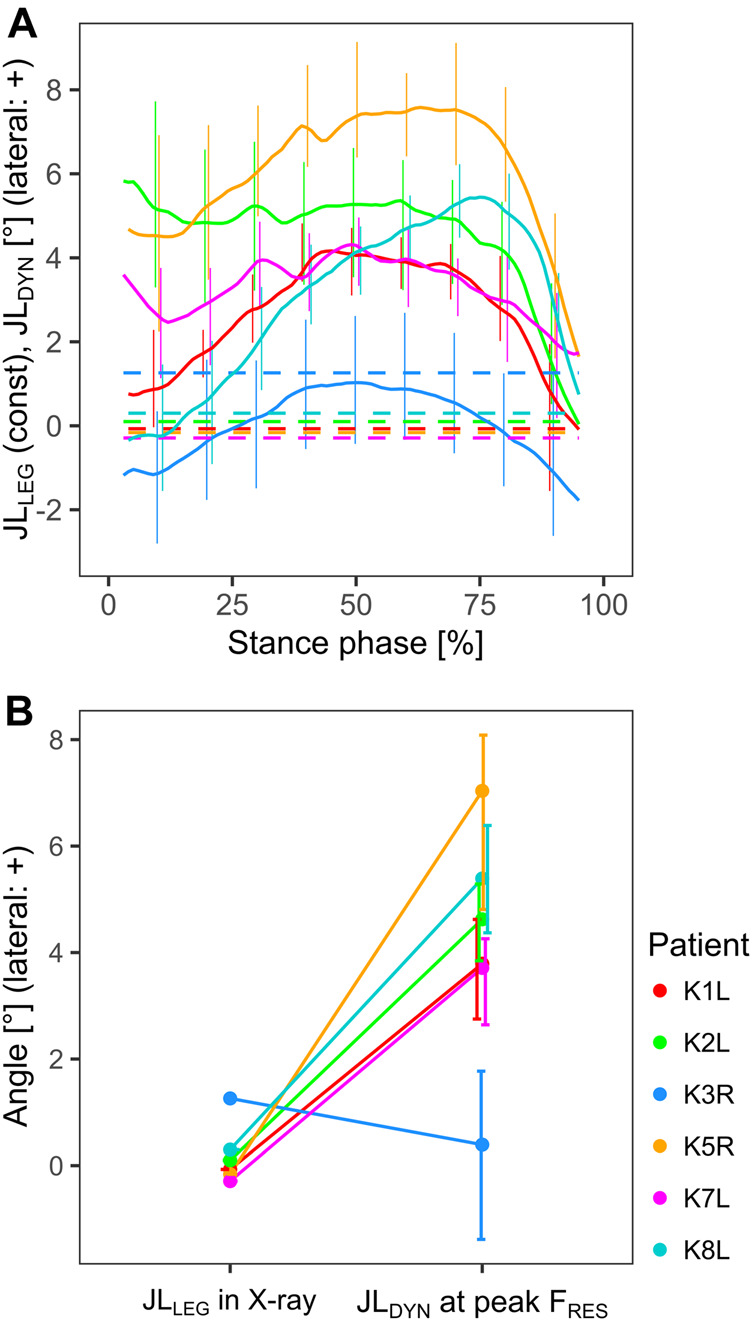
Comparison between the static joint line orientation relative to the orthogonal of the mechanical axis of the leg (JL_LEG_), and the dynamic joint line orientation relative to the ground (JL_DYN_), throughout the stance phase **(A)**, and at the time point of the peak tibio-femoral force **(B)**. Positive values indicate a tilt towards the lateral side.

During the stance phase of walking, the joint line was usually tilted laterally, while reaching maximal JL_DYN_ values of 5°–8° for most patients, except for K3R where it varied between 2° medial and 1° lateral tilt (Table 2; Figure 3A). At the time points of the peak resultant tibio-femoral contact force (F_RES_) and of the peak medial axial contact force (F_MED_) the JL_DYN_ was usually 0°–2° smaller than its maximal value (Table 2).

**TABLE 2:** Kinematics: The range of the dynamic lateral joint line tilt (JL_DYN_) during the stance phase of walking, and its value during peak F_RES_ and peak F_MED_ [mean (minimum, maximum)].

Patient	min JL_DYN_ [°]	Max JL_DYN_ [°]	JL_DYN_ at peak F_RES_ [°]	JL_DYN_ at peak F_MED_ [°]
K1L	−0.9 (−4.0, 0.7)	4.5 (4.2, 5.0)	3.8 (2.8, 4.6)	3.6 (2.4, 4.6)
K2L	−0.4 (−1.4, 1.0)	6.4 (3.8, 8.4)	4.6 (3.8, 5.3)	4.7 (3.4, 5.7)
K3R	−2.3 (−3.7, −0.3)	1.2 (−0.2, 2.8)	0.4 (−1.4, 1.8)	0.4 (−1.5, 1.7)
K5R	0.7 (−1.8, 2.5)	8.1 (6.5, 9.3)	7.0 (4.8, 8.1)	7.2 (4.8, 8.5)
K7L	1.3 (0.2, 2.8)	4.6 (3.6, 5.0)	3.7 (2.6, 4.3)	3.7 (2.6, 4.3)
K8L	−0.8 (−2.3, 0.4)	5.6 (5.0, 6.4)	5.4 (4.4, 6.4)	4.5 (1.8, 6.4)

The difference between the static joint line JL_LEG_ and the dynamic joint line JL_DYN_ during gait was patient specific, and in some patients quite substantial (Figure 3A). During peak F_RES_ the dynamic JL_DYN_ was tilted laterally by 4°–7° compared to the static JL_LEG_, except in patient K3R (Figure 3B).

### Static Alignment vs Joint Loading

The peak resultant tibio-femoral contact forces measured *in vivo* (F_RES_) varied substantially between the patients, with mean values ranging from 2.19 body-weight (BW) for K3R and 3.25 BW for K1L. The peak medial axial contact force (F_MED_) generally accounted for almost 80% of the corresponding total axial force, resulting in F_MED_ values between 1.72 BW for K3R, and 2.57 BW for K7L (Table 3).

**TABLE 3:** Loading: Peak internal resultant force (F_RES_), peak medial force (F_MED_) and the corresponding medial force ratio (MFR) and external adduction moment (EAM), as well as the peak F_MED_ during the first and second halves of the stance phase [mean (minimum, maximum)].

Patient	Peak F_RES_ [BW]	Peak F_MED_ [BW]	MFR at peak F_MED_ [-]	EAM at peak F_MED_ [%BWHt]	Early peak F_MED_ [BW]	Late peak F_MED_ [BW]
K1L	3.25 (2.92, 3.55)	2.45 (2.23, 2.68)	0.76 (0.73, 0.79)	2.96 (2.76, 3.19)	2.23 (1.97, 2.58)	2.43 (2.06, 2.68)
K2L	2.57 (2.38, 2.77)	2.01 (1.91, 2.15)	0.79 (0.77, 0.81)	3.22 (2.94, 3.38)	1.85 (1.74, 1.94)	2.01 (1.91, 2.15)
K3R	2.19 (1.98, 2.43)	1.72 (1.48, 1.87)	0.79 (0.76, 0.82)	2.67 (1.85, 3.37)	1.72 (1.48, 1.87)	1.51 (1.33, 1.74)
K5R	2.59 (2.28, 2.96)	1.83 (1.60, 2.03)	0.73 (0.60, 0.81)	1.98 (1.43, 2.60)	1.75 (1.41, 1.98)	1.78 (1.48, 2.03)
K7L	3.23 (2.98, 3.40)	2.57 (2.29, 2.80)	0.80 (0.75, 0.84)	3.69 (3.12, 4.39)	2.38 (2.09, 2.80)	2.57 (2.29, 2.79)
K8L	2.47 (2.24, 2.64)	1.94 (1.76, 2.11)	0.83 (0.78, 0.94)	2.96 (2.61, 3.33)	1.89 (1.69, 2.04)	1.91 (1.76, 2.11)

**FIGURE 4:**
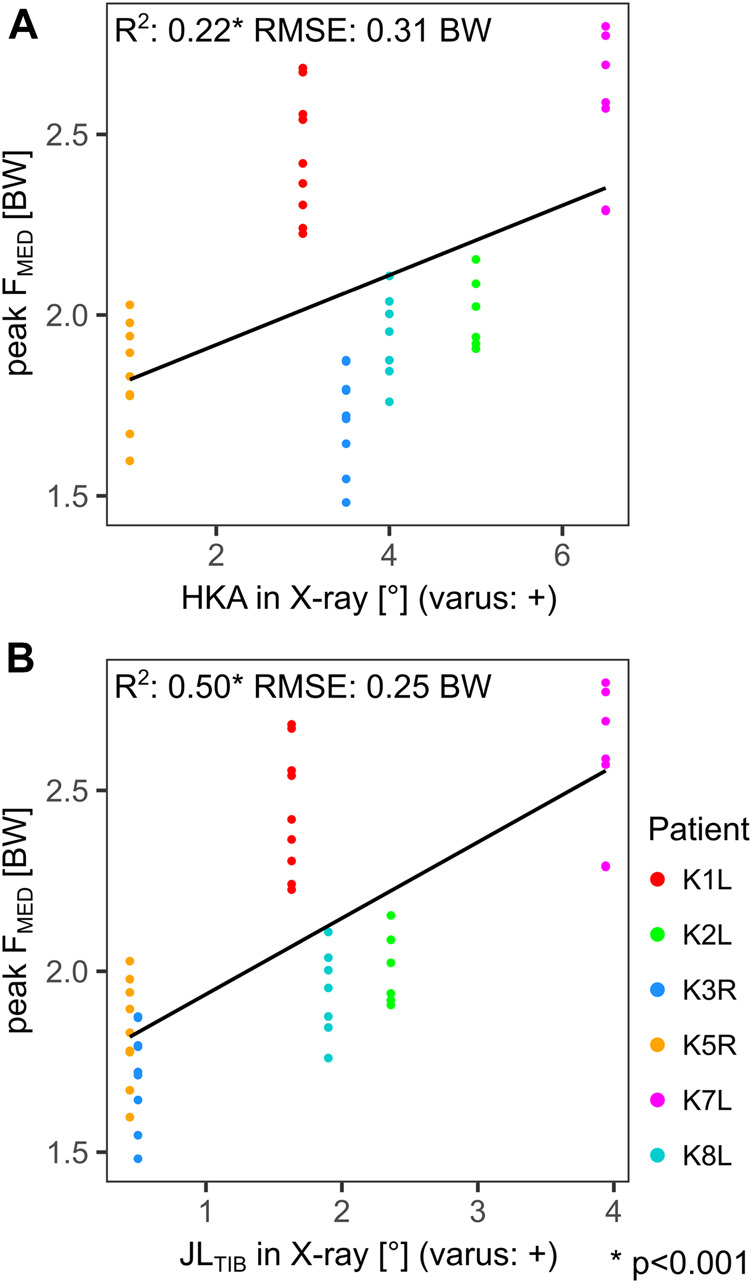
Linear regressions between the static hip-knee-ankle angle (HKA) **(A)**, the static tibial joint line (JL_TIB_) **(B)**, and the peak medial tibio-femoral force during gait (F_MED_). Each point represents a trial.

When the alignment from static frontal radiographs was compared to F_MED_, the strongest correlation was found to the JL_TIB_, with a coefficient of determination (*R* ^2^) of 0.50 and a root-mean-squared error (RMSE) of 0.25BW (Figure 4B). The correlation between peak F_MED_ and HKA was much weaker, with *R* ^2^: 0.22 and RMSE: 0.31BW (Figure 4A).

### Dynamic Alignment vs Joint Loading

**FIGURE 5:**
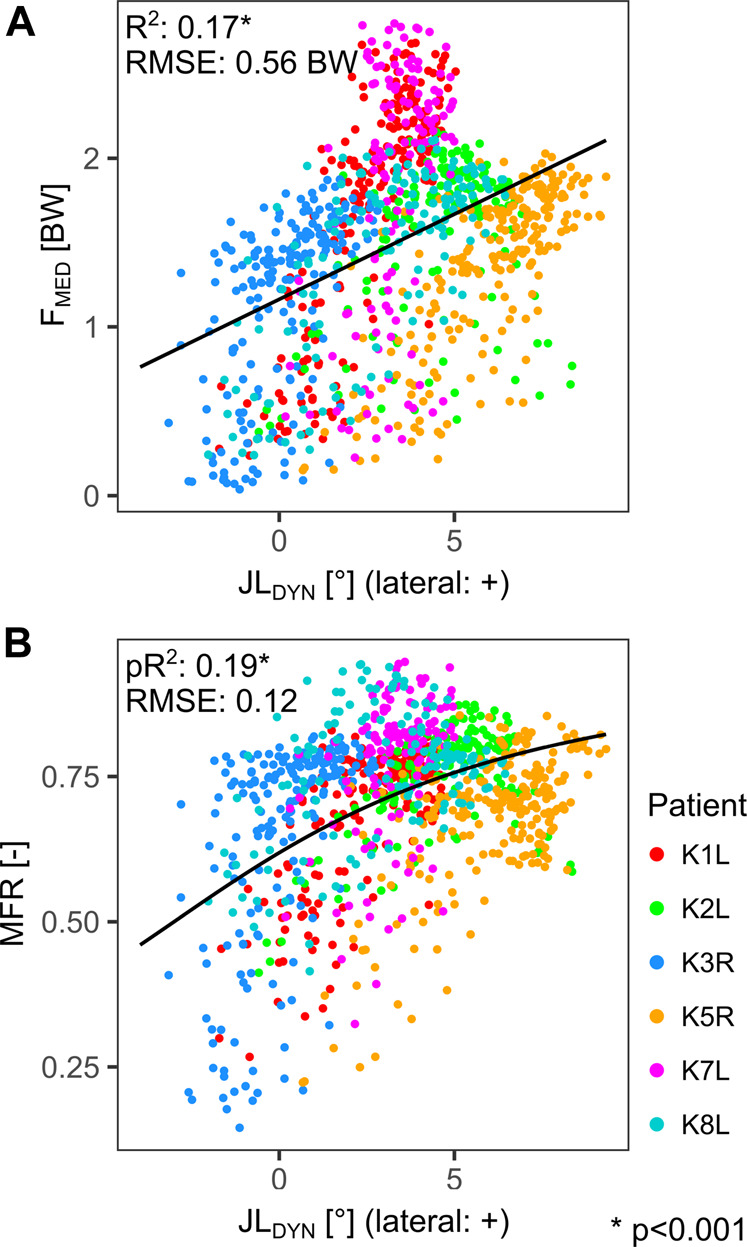
Linear regression between the dynamic joint line orientation relative to the ground (JL_DYN_) and the medial tibio-femoral force (F_MED_) **(A)**. Non-linear regression (arctan) between the dynamic joint line orientation relative to the ground (JL_DYN_) and the medial force ratio (MFR) **(B)**. Each point represents a time point during the stance phase.

When the stance phases of all patients were combined, the correlation between JL_DYN_ and F_MED_ was rather weak with an *R* ^2^ of 0.17 and a RMSE of 0.56 BW (Figure 5A). The non-linear model for the relation between JL_DYN_ and MFR yielded a pseudo *R* ^2^ of 0.19 and a RMSE of 0.12 (Figure 5B).

### External Loading vs Joint Loading

**FIGURE 6:**
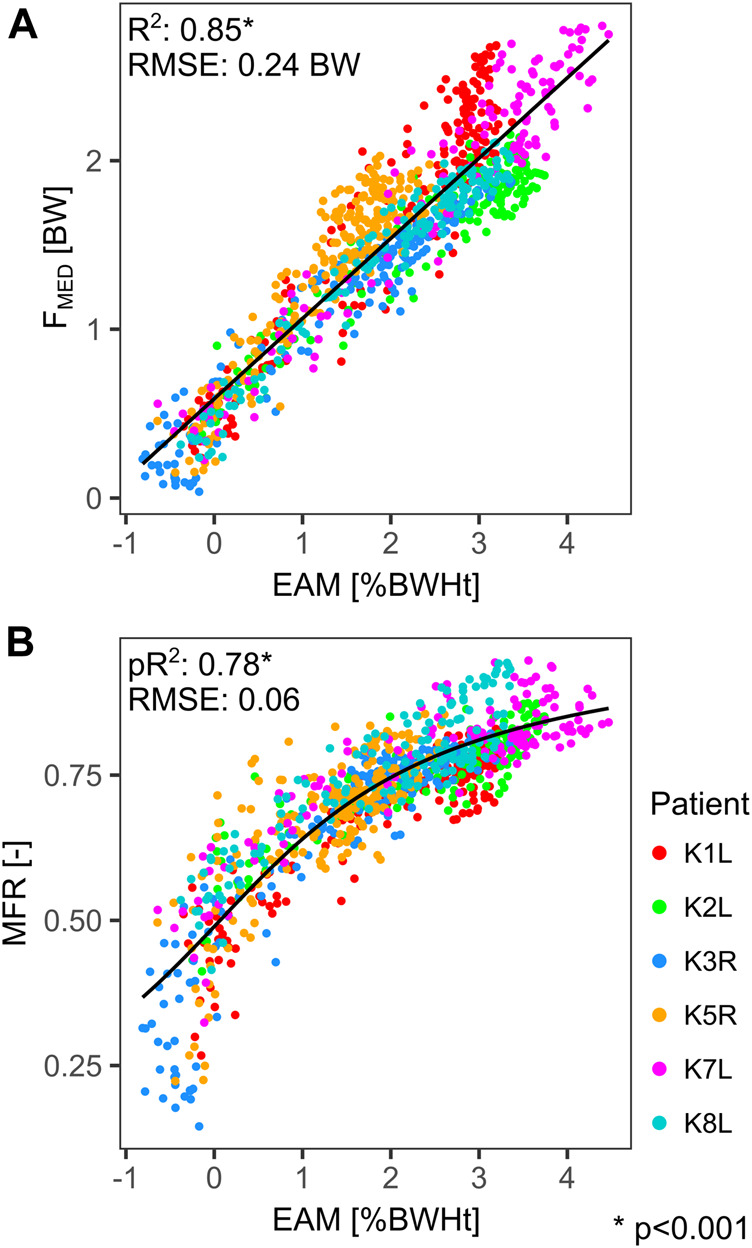
Linear regression between the external adduction moment (EAM) and the medial tibio-femoral force (F_MED_) **(A)**. Non-linear regression (arctan) between the external adduction moment (EAM) and the medial force ratio (MFR) **(B)**. Each point represents a time point during the stance phase.

**FIGURE 7:**
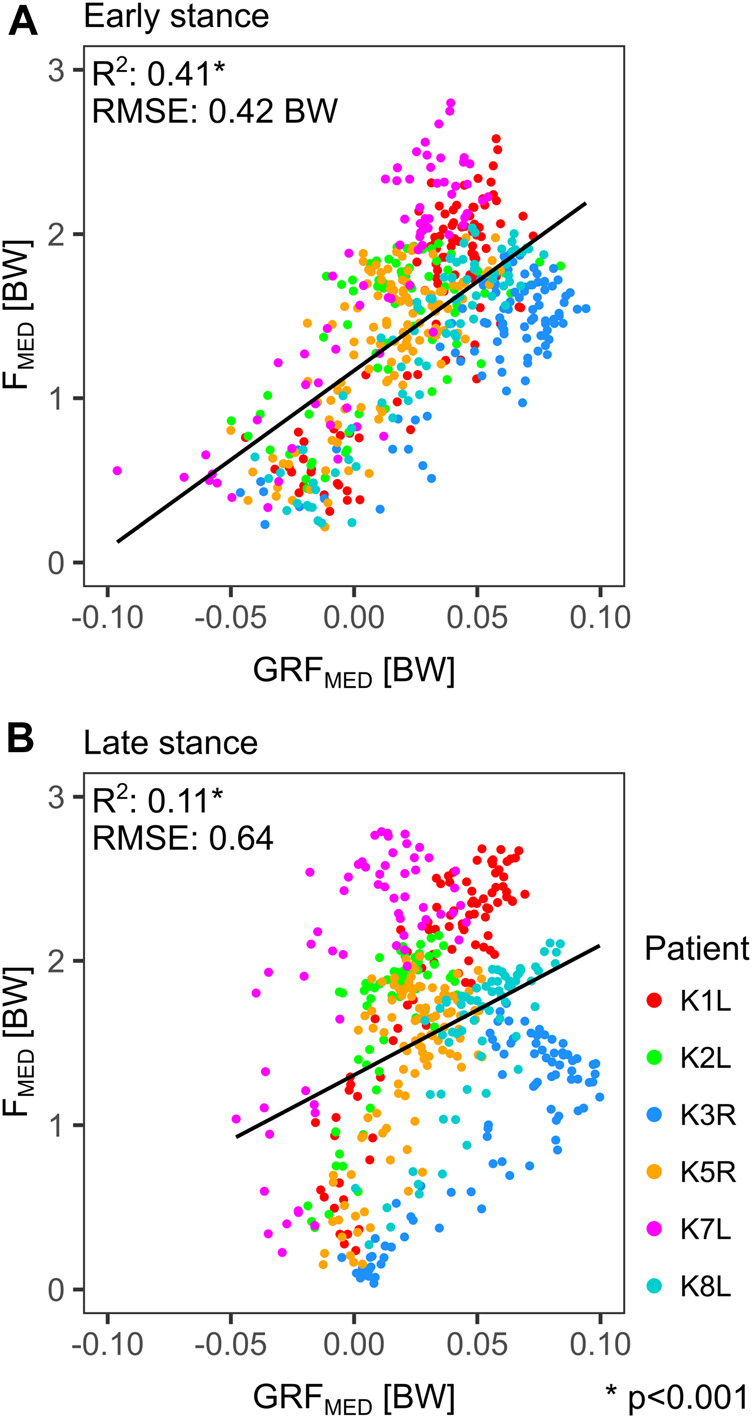
Linear regressions for all time points of the 1st **(A)** and 2nd half **(B)** of the stance phase during gait, between the medial component of the ground reaction force (GRF_MED_) and the medial knee contact force (F_MED_). Each point represents a time point during the stance phase.

In contrast to the dynamic alignment, the external adduction moment (EAM) showed strong correlations with F_MED_ throughout the stance phase of gait. For each individual patient, the regression between EAM and F_MED_ yielded *R* ^2^ values ranging from 0.85 (for K5R) to 0.96 (for K8L), while the RMSE ranged from 0.11 BW (for K8L) to 0.24 BW (for K1L). For all patients combined the *R* ^2^ was 0.85 and the RMSE was 0.24 BW (Figure 6A). The non-linear model for MFR also achieved a far better fit with EAM compared to the fit found in the JL_DYN_ analyses, with a pseudo *R* ^2^ of 0.78 and a RMSE of 0.06 (b_1_ = 0.50 %BWHt^−1^, b_2_ = 0.03) (Figure 6B). The vertical ground reaction force (GRF_SUP_) showed a clear correlation to F_MED_ in the early and late phase of stance with *R* ^2^ of 0.77 and 0.72 respectively. Although the medial ground reaction force (GRF_MED_) was only about 1/10th of the GRF_SUP_, it still explained 41% of the F_MED_ variance in the early stance phases, but was less relevant for F_MED_ in late stance (*R* ^2^ of 0.11) (Figure 7).

## Discussion

The tibial plateau of an intact knee joint is usually not orthogonal to the mechanical axis of the tibia (MA_TIB_), but rather tilted medially by 2°–3° in the frontal plane (varus) (Hungerford et al., 1982). When TKA is performed according to the surgical standard Mechanical Alignment Technique (MAT), a joint line orthogonal to MA_TIB_ is targeted, which here also implies orthogonality to the mechanical axis of the leg (MA_LEG_), since MA_TIB_ and MA_LEG_ are parallel in MAT. Alternative alignment techniques like Anatomical Alignment (AAT) and Kinematic Alignment (KAT) are aiming at a more physiological joint line orientation relative to the mechanical axes. Based on the assumption that it will lead to a more horizontal joint line during the single leg support phase of gait, AAT entails a 2°–3° joint line obliqueness relative to the mechanical axis of the leg (Hungerford et al., 1982) (Figure 1A), while KAT aims at reconstructing the patient’s preoperative anatomy, usually also leading to a physiological joint line obliqueness (Howell and Hull, 2014).

The differences between JL_LEG_ and JL_DYN_ observed in this study suggest that an oblique joint line, as implied by AAT and KAT, can indeed bring the dynamic joint line closer to a horizontal orientation during the stance phase of the gait in most patients. While this confirms our first hypothesis, it should be noted that the difference between JL_LEG_ and JL_DYN_ varied within each patient and showed substantial differences between patients (Figure 3A). One motivation for an oblique static joint line is the assumption that the resulting more horizontal dynamic joint line would lead to a more even medio-lateral distribution of contact forces in the knee joint and thus less straining on the medial condyle (Riviere et al., 2017a). Our results however do not show strong correlations between the dynamic joint line and the TF contact force distribution or with the medial TF contact force *in vivo* (Figure 5). Thus, our second hypothesis—which mirrors the philosophy of the oblique joint line—could not be confirmed.

The variable relationship between the static and the dynamic joint line during the loaded stance phase (Figure 3), and the weak correlation of static HKA to peak F_MED_ (Figure 4A), support the findings of other studies, that the static measures in frontal radiographs are not necessarily a casual predictor of the dynamic contact loads *in vivo* (Miller et al., 2014; Riviere et al., 2017b; Trepczynski et al., 2018). The static tibial joint line assessment JL_TIB_ had only moderate correlations to peak F_MED_ (Figure 4B).

In agreement with other studies (Kutzner et al., 2013; Trepczynski et al., 2014), the external adduction moment EAM proved to be a key determinant of the medial knee contact loads as well as for the contact load distribution (Figure 6). During the stance phase of walking, the EAM is mainly a result of the ground reaction force components in the frontal plane: the vertical GRF_SUP_ and the horizontal GRF_MED_. Despite being much smaller than GRF_SUP_, the GRF_MED_ component has a similar potential to generate considerable moments at the knee, due to its much longer lever arm. While the vertical GRF_SUP_ during walking is mainly weight and impact driven, muscle activity and gait patterns determine the medially oriented GRF_MED_, which accounts for 41% of the F_MED_ variance in the early stance phase (Figure 7A). Physiotherapy or preventive sports could therefore potentially modulate the muscle driven EAM component and thus help to reduce F_MED_.

It is currently not possible to compare *in vivo* measured knee loading across larger cohorts, with different TKA-designs and alignment approaches, so one has to be careful when generalizing the findings based on this unique cohort. Different implant designs are certainly known to allow varying tibio-femoral kinematics (Pfitzner et al., 2017; Schutz et al., 2019; Moewis et al., 2021; Postolka et al., 2021), but we believe that during the stance phase of level gait, the general relationships between frontal plane kinematics and the medio-lateral load distribution remain similar across most TKA-designs. Since knee flexion is limited in this situation, the different design approaches for stabilization during flexion play less of a role. It should also be noted that our cohort is biased towards varus alignment compared to the general TKA patient population (Luyckx et al., 2015; Almaawi et al., 2017), but such implantations are known to be associated with increased failure rates (Liu et al., 2016). While long term implant survivorship was not directly compared in this study, the loading of the medial condyle investigated here is likely to be relevant for medial bone collapse, which is a common tibial component failure mechanism (Berend et al., 2004), and for potential localized overloading of the PE inlay (Green et al., 2002).

Although the relatively small number of predominantly male patients and just one implant design are a limitation of this study, it still uses the worldwide largest cohort with synchronously measured *in vivo* forces, together with kinematics derived from gait assessment and mobile fluoroscopy. For the first time, this has allowed us to generate a reliable quantification of the relationships between internal and external loading and the kinematics of a TKA, as well as identification of the critical time points of maximal internal loading.

While implanting a TKA with a physiologically oblique joint line might have beneficial aspects, it does not appear to be essential for achieving an even load distribution across the condyles. Preventive und rehabilitative measures should focus on the EAM as an indicator of medial contact forces and consider the muscle driven component as a target for load modulating interventions.

## Data Availability

The data analyzed in this study is subject to the following licenses/restrictions: Restrictions apply to the availability of the implant geometry data, which were used under license for the current study, and so are not publicly available. Requests to access these datasets should be directed to; https://cams-knee.orthoload.com/data/data-download.
